# Patient preferences for the integration of mental health counseling and chronic disease care in South Africa

**DOI:** 10.2147/PPA.S176356

**Published:** 2018-09-18

**Authors:** Bronwyn Myers, John A Joska, Crick Lund, Naomi S Levitt, Christopher C Butler, Tracey Naledi, Peter Milligan, Dan J Stein, Katherine Sorsdahl

**Affiliations:** 1 Alcohol, Tobacco and Other Drug Research Unit, South African Medical Research Council, Cape Town, South Africa, bmyers@mrc.ac.za; 2 Division of Addiction Psychiatry, Psychiatry and Mental Health, bmyers@mrc.ac.za; 3 HIV and Mental Health Research Unit, Division of Neuropsychiatry, Department of Psychiatry and Mental Health; 4 Alan J Flisher Centre for Public Mental Health, Department of Psychiatry and Mental Health, University of Cape Town, Cape Town, South Africa; 5 Centre for Global Mental Health, Institute of Psychiatry, Psychology and Neuroscience, King’s College London, London, UK; 6 Division for Diabetes and Endocrinology, Department of Medicine, University of Cape Town, Cape Town, South Africa; 7 Nuffield Department of Primary Care Health Services, Oxford University, Oxford, UK; 8 Western Cape Department of Health, Cape Town, South Africa; 9 Faculty of Health Sciences, School of Public Health and Family Medicine, University of Cape Town, Cape Town, South Africa; 10 KwaZulu-Natal Department of Health, KwaZulu-Natal, South Africa; 11 Department of Psychiatry and Mental Health, University of Cape Town, Cape Town, South Africa; 12 SAMRC Unit on Anxiety and Stress Disorders, Cape Town, South Africa

**Keywords:** integration, mental health, chronic disease care, counseling preferences, primary health care, South Africa, global mental health, task sharing, alcohol, depression

## Abstract

**Purpose:**

To describe patient perceptions of the acceptability of integrating mental health counseling within primary care facilities in the Western Cape province of South Africa and their preferences for the way in which this care is delivered.

**Patients and methods:**

Qualitative interviews with 30 purposively selected patients receiving treatment for HIV or diabetes within primary care facilities who screened positive for depression using the Center for Epidemiological Studies Depression Scale or hazardous alcohol use through the Alcohol Use Disorders Identification Test.

**Results:**

Participants articulated high levels of unmet need for mental health services and strong associations between poor mental health and the challenges of living with a chronic disease. Consequently, they considered it acceptable to offer screening and mental health counseling within the context of chronic disease care. They thought counseling would be highly relevant if it helped patients develop adaptive strategies for coping with stress and negative emotions. Irrespective of chronic disease, patients indicated a preference for lay counselors rather than existing clinicians as potential delivery agents, supporting a task-shared approach to mental health counseling delivery in primary care settings. Some expressed concern about the feasibility of using lay counselors already present in facilities to deliver this service, suggesting that additional counselors might be needed.

**Conclusion:**

Findings demonstrate a need for mental health counseling within the context of chronic disease care in South Africa. Task-shared approaches, using lay counselors, seem acceptable to patients – provided counselors are selected to ensure they possess the qualities associated with effective counselors. Findings have informed the design of a task-shared mental health program that is responsive to the preferences of patients with chronic diseases.

## Introduction

Integrating mental health counseling into chronic disease services offered within primary health care (PHC) clinics has been widely recommended as a strategy for addressing chronic disease and mental health multimorbidity,^1^ reducing the substantial mental health treatment gap^2^ and enhancing the outcomes of chronic disease care in South Africa and other low- and middle-income countries (LMICs).^3^,^4^ While South African health policies support this strategy,^5^,^6^ prolonged shortages of mental health providers have limited the provision of integrated mental health and chronic disease care.^7^ To overcome these shortfalls and in line with the WHO guidelines for reducing the mental health treatment gap in LMICs,^8^ South Africa has endorsed task-sharing of basic mental health counseling from specialist mental health to nonspecialist health providers, such as nurses, lay counselors, and community health workers employed within PHC services.^6^ Evidence is accumulating that it is feasible to task-share mental health counseling within PHC services in LMICs and that task-shared interventions are potentially effective for treating depression, alcohol use, and other common mental disorders.^9^–^12^ Despite this evidence, it is not certain how chronic disease patients will perceive screening for alcohol problems and depression and the offer of counseling within a task-sharing framework.

There is concern that widespread provider stigma toward mental health and substance use problems,^13^–^16^ coupled with low recognition of the need for mental health interventions,^16^,^17^ may limit the uptake of mental health counseling by chronic disease patients. Earlier studies have described people with mental health, and substance use problems are often mistreated and denied chronic disease care due to misperceptions that their problems are a result of moral failing or beliefs that they are likely to be nonadherent. Consequently, patients are often reluctant to disclose their mental health and substance use concerns to health providers for fear of discriminatory treatment.^13^,^14^,^16^ As patient preferences for mental health services influence both the decision to initiate mental health care and the retention and outcomes of treatment,^18^–^21^ these preferences are important to consider when designing mental health services. In South Africa, limited involvement of patients in service planning has hampered the design of task-shared mental health services that are responsive to their needs and preferences.^22^ We are aware of only one other South African study that explored chronic disease patients’ preferences for mental health interventions. This study had a limited focus on alcohol-related needs among patients with HIV.^15^

To address this gap and optimize the design of a mental health counseling program potentially relevant to all chronic disease patients, we explored the mental health service needs and preferences of patients with chronic communicable (HIV) or noncommunicable diseases (diabetes) within PHC facilities in the Western Cape province of South Africa. This study aims to describe chronic disease patients’ perceptions of the acceptability of being offered mental health counseling within PHC facilities and their preferences for the way in which this counseling should be delivered.

## Methods

This manuscript complies with the Consolidated Criteria for Reporting Qualitative Research (COREQ).^23^

### Study design and setting

From September 2015 to March 2016, we interviewed patients receiving HIV or diabetes treatment at PHC clinics in the Western Cape province of South Africa. This was part of the formative work for project MIND, a cluster randomized controlled trial testing two approaches to integrating mental health counseling into chronic disease care.^24^ We interviewed patients from 17 purposively selected facilities that offer free basic health services to largely disadvantaged populations. These facilities varied in location, size, and resourcing, reflecting the heterogeneity present in this health care system.

### Participants and procedures

We approached patients waiting for their usual care appointment, described the study, and requested verbal consent to screen them for study inclusion. Fieldworkers administered a brief screening questionnaire to consenting patients to assess their eligibility for study inclusion. Patients were eligible if they were at least 18 years old, taking antiretroviral therapy for HIV or medication for diabetes, and scored ≥8 and ≤22 (reflecting hazardous/harmful alcohol use) on the Alcohol Use Disorders Identification Test^25^ or ≥16 on the Center for Epidemiological Studies Depression scale (CES-D), reflecting probable depression.^26^ Patients were excluded from participation if they were receiving treatment for a mental health condition. Maximum variation sampling was used to ensure the sample included males and females, patients with HIV, those with diabetes, those with alcohol use problems, and those with depression. Recruitment continued until the sample included participants with these characteristics and we reached saturation of content. Project staff invited eligible patients for an in-depth interview. All participants who scored positive for depression or hazardous alcohol use were provided feedback about their positive screen and offered referrals to mental health and substance use services within their community.

Trained male and female masters-level research assistants, experienced in conducting qualitative interviews, obtained participants’ written informed consent before beginning the interview. A semistructured interview guide elicited participants’ opinions about the acceptability of being asked questions about alcohol and depression and how they thought others might respond to this screening; the acceptability of offering mental health counseling to patients with chronic diseases, mental health counseling priorities, and preferences for counseling delivery. Interviews occurred in a private room in the participant’s choice of English, isiXhosa, or Afrikaans (official languages of the region). Interviews were audio-recorded before being transcribed verbatim and lasted up to 40 minutes. Where necessary, interview transcripts were translated into English using standard forward-back translation techniques. Participants were given refreshments, a grocery voucher for their time, and transport costs were reimbursed.

The South African Medical Research Council (EC 004-02/2015), the University of Cape Town (089/2015), and Oxford University (OxTREC 567-15) provided ethical approval for this study. The Western Cape Department of Health approved all procedures (WC 2015_RP 28-480).

### Analysis

We used the framework approach (familiarization, identifying a thematic framework, indexing, charting, mapping, and interpretation) for data analysis.^27^ Two study staff reviewed the transcripts, wrote memos for each transcript, identified emerging themes, and developed a coding frame. Working independently, they used NVivo version 11 to code the transcripts and met regularly to compare notes and resolve coding discrepancies. A third person was not needed to break coding ties. No new codes emerged after 16 transcripts were coded, suggesting thematic saturation. Intercoder reliability was high, with a Kappa score of 0.92.

## Results

Three broad themes emerged that reflect participants’ perceptions of the acceptability of the proposed counseling program and preferences for such services. The first theme describes participants’ views of how chronic disease patients will perceive screening and the offer of mental health counseling. The second theme describes potential targets for mental health counseling. The third theme describes participants’ preferences for the delivery of mental health counseling.

### Sample characteristics

The sample comprises 30 participants; 73% (23) were women. Participants’ mean age was 38.7 years (SD=13.4). Sixty-three percent (n=19) had HIV and 37% had diabetes (n=11). One participant had both conditions. Almost all participants scored above the CES-D cutoff for probable depression (M=37.1; SD=11.9). Seven participants (23%) were hazardous/harmful drinkers.

#### Acceptability of screening and counseling for mental health concerns

Overall, participants thought screening for depression and alcohol use within their usual chronic disease care visit was desirable as it could identify people who may benefit from counseling. They reflected that many people were unlikely to seek services by themselves due to “limited awareness and understanding” of mental health problems:

“It’s going to help you find out whether you have a problem, and if you have a problem to be assisted in time.” [Participant 23, HIV]

“It can be very helpful if they get screened because they regard themselves as well even though they are not […] they will actually discover the other problems that they didn’t know.” [Participant 5, Diabetes]

Although participants were generally supportive of this screening, several mentioned hesitancies about disclosing alcohol use due to concerns about health providers’ reactions:

“You say you are not drinking when you are […] in these clinics, some of us are asked questions about whether you are drinking. Then you will say no.” [Participant 9, HIV]

A few participants recommended that screening not take place at their treatment initiation visit, but rather after they have had time to absorb information related to their chronic disease:

“If it’s their first time to come here to the clinic, it won’t help, because they may be scared to talk. It will be better when they are already here, when they know everything about their chronic disease. Then you can do the screening.” [Participant 19, HIV]

Similarly, most participants thought offering mental health counseling as part of chronic disease care was acceptable, noting that a lot of people could benefit from counseling:

“There are many people [who] have problems. We are talking in the waiting area. There are many people that have things, situations like that.” [Participant 11, HIV]

Participants were realistic about whether chronic disease patients would accept an offer of mental health counseling, noting that “some will [accept] and some won’t.” For the most part, they thought that patients would welcome the opportunity (currently absent) “to talk about everything that bothers him with this counsellor.” They articulated how, if available, they would participate in counseling as they “yearned to make changes in their lives.”

### Mental health counseling needs

Participants thought mental health counseling should allow patients to “talk all their problems out” and “deal better with stress.” Coping with stress emerged as a key need; all participants described needing help coping with the stress of a chronic disease. Many participants spoke of struggling to accept their disease diagnosis which “mentally affected them.”

“I was lost in 2013, I didn’t accept it, so I just carried on with my lifestyle – drinking and smoking.” [Participant 13, HIV]

“A person no longer cares. This may be caused by not wanting to accept his health condition. Some even choose to die.” [Participant 2, Diabetes]

Some participants spoke of using alcohol to cope with “stress” and to “forget their problems” even though it “led to them not taking their medication.” Despite recognizing the impact of alcohol on medication adherence, several participants lacked motivation to reduce their drinking. Absence of hope and a sense of fatalism seemed to underpin limited motivation for change:

“Most of us when we drink alcohol we tell ourselves that we are doing it to reduce stress because we are living with HIV […] it’s better to drink alcohol to better the situation because I’m sick and I am going to die anyway.” [Participant 8, Diabetes and HIV]

Others described how difficulties in coping with stress led to excessive rumination and feelings of anger and depression (referred to as “negative thinking”):

“Thinking too much. You see as a person that always has problems, is always experiencing challenges … you are constantly thinking.” [Participant 15, HIV]

Most participants described how “negative thinking” made coping with their chronic disease more challenging, impacting on their concentration, remembering to take medication and energy to take charge of their health:

“You can even forget the time of taking your medication because you’ve got other things on your mind. If you think too much, you are not going to cope – it exhausts you and you can’t do anything.” [Participant 21, Diabetes]

### Counseling preferences

When asked about their preferences for how counseling should be structured and delivered, most were pragmatic and felt that they could benefit from “two to three” counseling sessions provided the service was flexible in terms of session scheduling to accommodate participants who were employed and could only attend the clinic once a month or on weekends:

“How is the counsellor going to accommodate those that are working and unavailable during the week? I believe weekends are appropriate to have something like this for people who are working.” [Participant 5, Diabetes]

“It doesn’t matter how many [sessions] there are […] all of us like me, farm people, we are allocated monthly clinic times so the counselling must also work this way.” [Participant 9, HIV]

When asked about who they would like to deliver counseling, there was agreement among participants that doctors and nurses should not provide counseling. Irrespective of chronic disease diagnosis, participants reported being “scared” of these providers and did “not have confidence in their ability” to conduct counseling. There was consensus that lay counselors responsible for conducting adherence counseling were best suited to deliver this service:

“Not the nurses […] when I have a problem I must talk with my counsellor. You can’t talk with the nurses. They make you feel like a nobody. The nurse is not the same as the counsellor.” [Patient 11, HIV]

Participants were divided over whether it was feasible and acceptable for their current adherence counselor to deliver mental health care or whether a new “specialist” lay counselor responsible solely for the delivery of this service was needed. Those who had a good relationship with their current counselor generally thought that this counselor’s role should expand to include the new service:

“It would be better if it can be the person that we are all familiar with as they know how to approach us. People can easily open up because they know this person.” [Participant 17, HIV]

However, some expressed concern that their counselor was “already busy and may not have time” to deliver an extra service. They thought that it may be more feasible to have an additional counselor deliver mental health counseling, noting this may limit patient waiting time and improve the quality of care:

“They have got a lot of work to do […] Adding more counsellors will make their work a little easier. Then the counsellors don’t have to rush when they give you counselling.” [Participant 13, HIV]

Regardless of chronic disease diagnosis, participants who had more negative views of their current counselors felt that additional “specialist” counselors were required for mental health counseling to be acceptable and impactful. These participants were concerned about the limited professional ethics and lack of confidentiality among current lay counselors, which they thought would impact on patient participation:

“Everyone knows each other and so you can’t really empty yourself […] you will let go a little, but you will always apply brakes.” [Participant 20, Diabetes]

These participants wanted lay counselors responsible for mental health counseling to have the following characteristics: “humble,” “respectful,” “approachable,” “professional,” and “trained” so that patients “feel free to talk from day one.” As one participant reflected:

“Don’t just take someone who is here to earn, but someone who has a passion for what they are doing. They must have a way to talk to people, because there are some people who don’t have a way.” [Participant 19, HIV]

## Discussion

Despite global initiatives to integrate mental health counseling into chronic disease services,^4^ patients’ preferences for such services have not been fully examined. This study begins to address this gap by exploring the patients’ preferences of South African chronic disease for this service. Findings suggest that, irrespective of chronic disease diagnosis, participants thought 1) it largely acceptable to offer screening and mental health counseling within the context of chronic disease care; 2) the relevance of counseling could be enhanced by helping patients develop adaptive strategies for coping with stress; and 3) it identified lay counselors as preferred mental health counseling delivery agents, provided they were competent and had the requisite attributes.

Given high levels of unmet need for mental health support among chronic disease patients, a recognition that mental health concerns were closely interlinked with the challenges of having a chronic disease and acknowledgement that poor mental health affected treatment adherence, participants generally thought it acceptable to screen and provide counseling for mental health problems within the context of chronic disease care. Nonetheless and in line with other studies,^15^,^28^ they recognized that low mental health literacy and widespread stigma surrounding mental disorders in general and alcohol problems in particular may impact the patients’ responses to screening and the offer of counseling. The timing of screening may be critical for optimal patient participation in this new service. Participants thought patients would be more likely to consider screening and counseling if it is offered after treatment initiation as patients would have had more time to adjust to their chronic disease diagnosis. Based on this feedback, we have incorporated mental health awareness-raising activities at all PHC clinics implementing project MIND. We have also adjusted our protocols so that mental health screening occurs after treatment initiation and that patients are provided with multiple opportunities for entering the program in case they initially decline counseling.

Participants identified potential psychological and behavioral priority areas for mental health counseling that could enhance counseling relevance for this patient population. These include needs related to motivation for changing unhealthy behaviors, resolution of life problems and stressors, management of negative emotions, and acceptance of their chronic disease. These findings suggest that counseling that includes motivational components and problem-solving therapy (a structured form of cognitive-behavioral therapy that not only builds skills for resolving mutable problems but also enhances emotion-focused coping for immutable problems) may be helpful in this context.^29^–^32^ The combination of motivational interviewing components with PST has been found effective for reducing substance use and depression in other South African patient populations.^32^ Other studies have shown that manualized interventions utilizing this combined approach seem feasible and acceptable to task-shift to lay counselors.^33^,^34^ Our findings suggest that the relevance and acceptability of this combined approach may extend to patients living with chronic diseases. Regardless of the counseling approach adopted, our findings of widespread social isolation and lack of support suggest that any mental health program should encourage patients to seek additional support and raise awareness of where they might obtain this support in their community.

Next, patients were more concerned about the choice of counseling agent than counseling format. Their preference for lay counselors as intervention agents reflects the acceptability of task-sharing mental health counseling with these cadres of health workers. About half of the participants were satisfied with their current lay counselor delivering this service due to established rapport and continuity of care. Others expressed concern about the capacity of existing lay counselors to provide new services and concerns about professionalism, competence, and interest in mental health. Previous studies outlined similar misgivings, albeit from a provider and system perspective.^6^,^9^,^35^ To ensure the feasibility and acceptability of this new service, these participants recommended the addition of new counselors trained, supervised, and competent to provide mental health counseling. Participants’ lack of consensus on this issue reflects broader uncertainty about whether it is more feasible and effective to use “specialist” lay counselors dedicated to the delivery of mental health care or more efficient to use existing counselors designated to deliver this new service in addition to their usual tasks.

Findings should be considered in the light of some limitations. The sample was small; participants may not have been representative of all patients receiving chronic disease care. Related to this, almost three-quarters of the sample were women. While this reflects the well-documented gender disparities in PHC utilization,^1^,^36^ future studies should strive to include equal proportions of male and female participants, particularly as men are more likely to have hazardous patterns of alcohol use.^2^ The degree to which these findings can be generalized to provinces other than the Western Cape is unclear. Importantly, the sample excluded patients in emerging adulthood and adolescents. While our sample reflects the demographic profile of HIV and other chronic disease services in South Africa,^1^,^36^ with younger people reluctant to utilize adult-oriented services,^37^ it is a cause for concern as young people are particularly vulnerable to mental health and substance use problems.^38^ Future studies should consider how to expand the mental health counseling services available in PHC settings to other settings where young people are likely to seek health and social care. Nonetheless, despite some limitations, this qualitative study helped identify factors critical to consider when designing patient-centered mental health services.

## Conclusion

Despite some limitations, this study provides insights into chronic disease patients’ preferences for mental health counseling. The study is novel, in that it explores the preferences of patients with a chronic communicable and those with a noncommunicable disease. Findings demonstrate a need for mental health counseling within the context of chronic disease care in South Africa, highlighting the importance of investing in this service. As patients with communicable and noncommunicable diseases indicated similar mental health service needs and preferences, tailoring mental health counseling to different types of chronic diseases may not be required. Task-shared approaches, using lay counselors, seem acceptable to patients – provided counselors are selected to ensure they possess the qualities associated with effective counselors and trained for competence. As patients had diverging views on the desirability of a dedicated (“specialist”) counselor or someone who integrates mental health counseling into their existing roles, further research is needed to explore the acceptability and effectiveness of these approaches to the delivery of task-shared mental health counseling within chronic disease services.

## References

[1] Folb N, Timmerman V, Levitt N (2015). Multi-morbidity, control and treatment of non-communicable diseases among primary healthcare attenders in the Western Cape, South Africa. S Afr Med J.

[2] Seedat S, Stein DJ, Herman A (2008). Twelve-month treatment of psychiatric disorders in the South African Stress and Health Study (World Mental Health Survey Initiative). Soc Psychiatry Psychiatr Epidemiol.

[3] Shidhaye S, Lund C, Chisholm D (2015). Closing the treatment gap for mental, neurological and substance use disorders by strengthening existing health care platforms: strategies for delivery and integration of evidence-based interventions. Int J Ment Health Syst.

[4] Ngo VK, Rubinstein A, Ganju V (2013). Grand challenges: integrating mental health care into the non-communicable disease agenda. PLoS Med.

[5] Mahomed OH, Asmall S, Freeman M (2014). An integrated chronic disease management model: a diagonal approach to health system strengthening in South Africa. J Health Care Poor Underserved.

[6] South African National Department of Health (2013). National Mental Health Policy Framework and Strategic Plan, 2013–2020.

[7] Petersen I, Marais D, Abdulmalik J (2017). Strengthening mental health system governance in six low- and middle-income countries in Africa and South Asia: challenges, needs and potential strategies. Health Policy Plan.

[8] World Health Organization (2013). Mental Health Action Plan 2013–2020.

[9] Padmanathan P, De Silva MJ (2013). The acceptability and feasibility of task-sharing for mental healthcare in low- and middle-income countries: a systematic review. Soc Sci Med.

[10] Singla DR, Kohrt BA, Murray LK (2017). Psychological treatments for the world: lessons from low-and middle-income countries. Annu Rev Clin Psychol.

[11] van Ginneken N, Tharyan P, Lewin S (2013). Non-specialist health worker interventions for the care of mental, neurological and substance-abuse disorders in low- and middle-income countries. Cochrane Database Syst Rev.

[12] Hoeft TJ, Fortney JC, Patel V (2018). Task-sharing approaches to improve mental health care in rural and other low-resource settings: a systematic review. J Rural Health.

[13] Sorsdahl KR, Stein DJ (2010). Knowledge of and stigma associated with mental disorders in a South African community sample. J Nerv Mental Dis.

[14] Mall S, Sorsdahl K, Struthers H (2013). Mental health in primary human immunodeficiency virus care in South Africa: a study of provider knowledge, attitudes, and practice. J Nerv Mental Dis.

[15] Kekwaletswe CT, Morojele NK (2014). Alcohol use, antiretroviral therapy adherence, and preferences regarding an alcohol-focused adherence intervention in patients with human immunodeficiency virus. Patient Prefer Adherence.

[16] Myers B, Carney T, Wechsberg WM (2016). “Not on the agenda”: a qualitative study of influences on health services use among poor young women who use drugs in Cape Town, South Africa. Int J Drug Policy.

[17] Myers B, Van Der Westhuizen C, Naledi T (2016). Readiness to change is a predictor of reduced substance use involvement: findings from a randomized controlled trial of patients attending South African emergency departments. BMC Psychiatry.

[18] Goodrich DE, Kilbourne AM, Nord K (2013). Mental health collaborative care and its role in primary care settings. Curr Psychiatry Rep.

[19] Swift JK, Callahan JL (2009). The impact of client treatment preferences on outcome: a meta-analysis. J Clin Psychol.

[20] Swift JK, Callahan JL, Vollmer BM (2011). Preferences. J Clin Psychol.

[21] King M, Nazareth I, Lampe F (2005). Impact of participant and physician intervention preferences on randomized trials: a systematic review. JAMA.

[22] Kleintjes S, Lund C, Swartz L (2013). Barriers to the participation of people with psychosocial disability in mental health policy development in South Africa: a qualitative study of perspectives of policy makers, professionals, religious leaders and academics. BMC Int Health Hum Rights.

[23] Tong A, Sainsbury P, Craig J (2007). Consolidated criteria for reporting qualitative research (COREQ): a 32-item checklist for interviews and focus groups. Int J Qual Health Care.

[24] Myers B, Lund C, Lombard C (2018). Comparing dedicated and designated models of integrating mental health into chronic disease care: study protocol for a cluster randomized controlled trial. Trials.

[25] Babor T, Higgins-Biddle J, Saunders J (2001). The Alcohol Use Disorders Identification Test, Guidelines for Use in Primary Care. World Health Organization: Department of Mental Health and Substance Dependence.

[26] Lewinsohn PM, Seeley JR, Roberts RE, Allen NB (1997). Center for Epidemiological Studies-Depression Scale (CES-D) as a screening instrument for depression among community-residing older adults. Psychol Aging.

[27] Ritchie J, Spencer L, Bryman A, Burgess RG (1994). Qualitative data analysis for applied policy research. Analysing Qualitative Data.

[28] Sorsdahl KR, Mall S, Stein DJ, Joska JR (2010). Perspectives towards mental illness in people living with HIV/AIDS in South Africa. AIDS Care.

[29] Sorsdahl K, Myers B, Ward CL (2015). Adapting a blended motivational interviewing and problem-solving intervention to address risky substance use amongst South Africans. Psychother Res.

[30] Malouff JM, Thorsteinsson EB, Schutte NS (2007). The efficacy of problem solving therapy in reducing mental and physical health problems: a meta-analysis. Clin Psychol Rev.

[31] Myers B, Sorsdahl K, Morojele NK, Kekwaletswe C, Shuper PA, Parry CD (2016). “In this I have everything I need”: perceived acceptability of a brief alcohol-focused intervention for people living with HIV. AIDS Care.

[32] Sorsdahl K, Stein DJ, Corrigall J (2015). The efficacy of a blended motivational interviewing and problem solving therapy intervention to reduce substance use among patients presenting for emergency services in South Africa: A randomized controlled trial. Subst Abuse Treat Prev Policy.

[33] Sorsdahl K, Petersen Williams P, Everett-Murphy K (2015). Feasibility and preliminary responses to a screening and brief intervention program for maternal mental disorders within the context of primary care. Community Ment Health J.

[34] Sorsdahl K, Myers B, Ward C (2014). Screening and brief interventions for substance use in emergency departments in the Western Cape province of South Africa: views of health care professionals. Int J Inj Contr Saf Promot.

[35] Petersen I, Fairall L, Egbe CO, Bhana A (2014). Optimising lay counsellor services for chronic care in South Africa: a qualitative systematic review. Patient Educ Couns.

[36] Nteta TP, Mokgatle-Nthabu M, Oguntibeju OO (2010). Utilization of the primary health care services in the Tshwane Region of Gauteng Province, South Africa. PLoS One.

[37] Geary RS, Webb EL, Clarke L, Norris SA (2015). Evaluating youth-friendly health services: young people’s perspectives from a simulated client study in urban South Africa. Glob Health Action.

[38] Nagata JM, Hathi S, Ferguson BJ, Hindin MJ, Yoshida S, Ross DA (2018). Research priorities for adolescent health in low- and middle-income countries: a mixed-methods synthesis of two separate exercises. J Glob Health.

